# Outpatient Antibiotic Prescribing for Acute Respiratory Infections During Influenza Seasons

**DOI:** 10.1001/jamanetworkopen.2018.0243

**Published:** 2018-06-08

**Authors:** Fiona P. Havers, Lauri A. Hicks, Jessie R. Chung, Manjusha Gaglani, Kempapura Murthy, Richard K. Zimmerman, Lisa A. Jackson, Joshua G. Petrie, Huong Q. McLean, Mary Patricia Nowalk, Michael L. Jackson, Arnold S. Monto, Edward A. Belongia, Brendan Flannery, Alicia M. Fry

**Affiliations:** 1 Centers for Disease Control and Prevention, Atlanta, Georgia; 2 Baylor Scott & White Health, Texas A&M University Health Science Center College of Medicine, Temple; 3 University of Pittsburgh, Pittsburgh, Pennsylvania; 4 Kaiser Permanente Washington Health Research Institute, Seattle; 5 Department of Epidemiology, University of Michigan School of Public Health, Ann Arbor; 6 Marshfield Clinic Research Foundation, Marshfield, Wisconsin

## Abstract

**Importance:**

Acute respiratory infections (ARIs) are the syndrome for which antibiotics are most commonly prescribed; viruses for which antibiotics are ineffective cause most ARIs.

**Objectives:**

To characterize antibiotic prescribing among outpatients with ARI during influenza season and to identify targets for reducing inappropriate antibiotic prescribing for common ARI diagnoses, including among outpatients with laboratory-confirmed influenza.

**Design, Setting, and Participants:**

Cohort study enrolling outpatients aged 6 months or older with ARI evaluated at outpatient clinics associated with 5 US Influenza Vaccine Effectiveness Network sites during the 2013-2014 and 2014-2015 influenza seasons. All patients received influenza testing by real-time reverse transcriptase–polymerase chain reaction for research purposes only. Antibiotic prescriptions, medical history, and *International Classification of Diseases, Ninth Revision* diagnosis codes were collected from medical and pharmacy records, as were group A streptococcal (GAS) testing results in a patient subset.

**Exposure:**

Visit for ARI, defined by a new cough of 7 days’ duration or less.

**Main Outcomes and Measures:**

Antibiotic prescription within 7 days of enrollment. Appropriateness of antibiotic prescribing was based on diagnosis codes, clinical information, and influenza and GAS testing results.

**Results:**

Of 14 987 patients with ARI (mean [SD] age, 32 [24] years; 8638 [58%] women; 11 892 [80%] white), 6136 (41%) were prescribed an antibiotic. Among these 6136 patients, 2522 (41%) had diagnoses for which antibiotics are not indicated; 2106 (84%) of these patients were diagnosed as having a viral upper respiratory tract infection or bronchitis (acute or not otherwise specified). Among the 3306 patients (22%) not diagnosed as having pneumonia and who had laboratory-confirmed influenza, 945 (29%) were prescribed an antibiotic, accounting for 17% of all antibiotic prescriptions among patients with nonpneumonia ARI. Among 1248 patients with pharyngitis, 1137 (91%) had GAS testing; 440 of the 1248 patients (35%) were prescribed antibiotics, among whom 168 (38%) had negative results on GAS testing. Of 1200 patients with sinusitis and no other indication for antibiotic treatment who received an antibiotic, 454 (38%) had symptoms for 3 days or less prior to the outpatient visit, suggesting acute viral sinusitis not requiring antibiotics.

**Conclusions and Relevance:**

Antibiotic overuse remains widespread in the treatment of outpatient ARIs, including among patients with laboratory-confirmed influenza, although study sites may not be representative of other outpatient settings. Identified targets for improved outpatient antibiotic stewardship include eliminating antibiotic treatment of viral upper respiratory tract infections and bronchitis and improving adherence to prescribing guidelines for pharyngitis and sinusitis. Increased access to sensitive and timely virus diagnostic tests, particularly for influenza, may reduce unnecessary antibiotic use for these syndromes.

## Key Points

**Question:**

What are targets for improving antibiotic stewardship for outpatient acute respiratory infections?

**Findings:**

Among 14 987 outpatients with acute respiratory infections enrolled in this cohort study during influenza seasons, 41% were prescribed antibiotics, 41% of whom had diagnoses for which antibiotics are not indicated, primarily viral upper respiratory tract infections and bronchitis; 29% of patients with influenza confirmed through research testing were prescribed antibiotics. Among patients prescribed antibiotics, 38% with pharyngitis tested negative for group A streptococcus and 38% with sinusitis had symptoms for 3 days or less before the visit, suggesting antibiotic therapy was not required.

**Meaning:**

Eliminating antibiotic treatment of viral upper respiratory tract infections and bronchitis, improving influenza diagnosis and treatment, and reinforcing prescription guidelines for pharyngitis and sinusitis could improve outpatient antibiotic stewardship.

## Teaser

This cohort study examines patterns and appropriateness of antibiotic prescribing among children and adults with acute respiratory infections visiting outpatient clinics associated with the US Influenza Vaccine Effectiveness Network during influenza season.

## Introduction

Acute respiratory infections (ARIs) remain the clinical category for which antibiotics are most commonly prescribed.^1,2^ However, most ARIs are caused by viruses for which antibiotics have no role in treatment. Inappropriate antibiotic use contributes to the development of antibiotic-resistant organisms, which cause an estimated 2 million illnesses and 23 000 deaths annually in the United States.^3^ Understanding antibiotic prescribing practices for ARIs in outpatient settings is critical to designing strategies for reducing inappropriate antibiotic use.

Previous studies examining antibiotic overuse have relied on national survey data, and many lack clinical and laboratory testing results.^2,4,5,6,7^ In this study, we include data on illness onset date, laboratory testing for influenza in all patients, and, for many patients, self-reported fever and the results of clinician-ordered group A streptococcal (GAS) testing. We used 3 approaches to assess antibiotic prescribing among outpatients with ARIs during the influenza season. First, we examined antibiotic prescribing by age group, antibiotic type, and diagnosis. Second, because influenza is a frequent cause of ARIs, sensitive point-of-care influenza testing is not widely available,^8,9^ and individuals with influenza are often inappropriately prescribed antibiotics,^10^ we characterized antibiotic prescribing among those with laboratory-confirmed influenza, excluding syndromes for which antibiotics are indicated (eg, pneumonia). Third, we examined appropriateness of antibiotic prescribing for those with pharyngitis, sinusitis, and otitis media (OM), diagnoses that account for nearly one-third of prescribed outpatient antibiotics,^2^ based on symptom duration, presence of fever, prescription of recommended first-line antibiotics,^4^ and GAS testing results.

## Methods

### Enrollment

In this cohort study, we used data collected by the US Influenza Vaccine Effectiveness Network during the 2013-2014 (defined here as December 2, 2013, through April 16, 2014) and 2014-2015 (defined here as November 10, 2014, through April 10, 2015) influenza seasons, as described elsewhere.^11^ The network, which included 57 (2013-2014) and 66 (2014-2015) clinics associated with 5 sites that are geographically diverse academic medical centers and health care organizations, was designed to assess influenza vaccine effectiveness annually; additional data were collected for this secondary analysis. Once sites confirmed local influenza circulation, patients aged 6 months or older with ARI, defined by a new cough of 7 days’ duration or less, were eligible for enrollment. Respiratory swab specimens were obtained and tested for influenza with real-time reverse transcriptase–polymerase chain reaction for research purposes only. Patients could enroll more than once per season more than 14 days apart; we analyzed each patient visit separately. Eligible patients were screened and enrolled if they or their legal guardians provided written or oral informed consent, depending on requirements of local institutional review boards. Study procedures, forms, and consent documents were approved by site institutional review boards.

Patient characteristics, including self-reported race and ethnicity selected from options defined by the investigator, and symptom onset date were ascertained by interview. In addition, patients were asked about self-reported fever at 2 sites in 2013-2014 and all sites in 2014-2015. We obtained patients’ history of underlying medical conditions by examining *International Classification of Diseases, Ninth Revision* (*ICD-9*) codes assigned to medical encounters during the year before enrollment. We extracted dates of hospitalizations occurring less than 30 days after enrollment from medical records. Excluding topical antibiotics, we obtained antibiotic and influenza antiviral prescriptions within 7 days of enrollment from pharmacy, insurance, or electronic medical records. Patients were excluded if dates indicated antibiotics were first prescribed during a hospitalization. If more than 1 antibiotic was prescribed, the antibiotic with the earliest prescription date was included.

### Diagnostic Categories

We classified the first 4 *ICD-9* codes assigned to the enrollment visit into 3 tiers based on previously published classifications (eTable 1 in the Supplement). Tier 1 includes diagnoses for which antibiotics are almost always indicated, including pneumonia; per Infectious Diseases Society of America/American Thoracic Society guidelines, empirical antibiotic treatment is appropriate for persons with pneumonia, regardless of the etiology.^12^ Tier 2 includes diagnoses for which antibiotics may be indicated, eg, sinusitis, pharyngitis, and suppurative OM. Tier 3 includes all other diagnoses for which antibiotics are not indicated or the indication was unclear; these include viral upper respiratory tract infection (URI), influenza, allergy, asthma, and acute bronchitis.^2^ Because current guidance recommends antibiotic treatment for some patients with chronic obstructive pulmonary disease (COPD) exacerbations,^13^ adults with a history of COPD or COPD diagnosis code at enrollment were excluded from bronchitis analyses. We excluded patients missing *ICD-9* codes or assigned diagnostic codes compatible with nonrespiratory diagnoses for which antibiotics are potentially indicated (eg, urinary tract infection). If a patient had multiple diagnoses, priority was given to tier 1 diagnoses, then tier 2 diagnoses, then tier 3 diagnoses.

### Antibiotic Classification

We classified antibiotics based on previously established categories.^14^ Narrow-spectrum antibiotics included narrow-spectrum penicillins, tetracyclines, first-generation cephalosporins, and sulfonamides. Broad-spectrum antibiotics included macrolides (eg, azithromycin), broad-spectrum penicillins (eg, amoxicillin-clavulanate), advanced-generation cephalosporins, quinolones, and lincomycin derivatives (clindamycin). We examined whether clinicians prescribed first-line antibiotics for the most common tier 2 diagnoses, based on national guidelines: penicillin or amoxicillin for pharyngitis, amoxicillin or amoxicillin-clavulanate for sinusitis, and amoxicillin or amoxicillin-clavulanate (alternative) for suppurative OM.^4,15,16,17,18,19^

### Influenza Testing

At 1 site, clinicians were provided study real-time reverse transcriptase–polymerase chain reaction results for influenza within 48 hours of enrollment. Results were not available to clinicians at other sites. At 4 sites, we examined data on rapid influenza diagnostic tests or other diagnostic tests ordered the day of enrollment and not performed as part of the study protocol.

### Pharyngitis and GAS Testing

For pharyngitis, guidelines recommend antibiotic therapy only for GAS pharyngitis and diagnostic testing if GAS is suspected.^20^ We examined a GAS testing subset comprising patients with a pharyngitis diagnosis with no other diagnosis for which antibiotics may be warranted at 4 sites during the 2014-2015 season for whom we obtained information from medical records on clinician-ordered GAS testing. Rapid antigen detection test results were available at all sites, and some sites performed cultures when rapid antigen detection test results were negative. If a patient had positive results on either the GAS rapid antigen detection test or culture, the patient was considered GAS positive. The proportion of patients in the entire study population with pharyngitis who were prescribed an antibiotic despite a negative result on GAS testing was estimated based on results from the GAS testing subset.

### Sinusitis and OM

For sinusitis, national guidelines recommend antibiotics for adults and children with severe symptoms, defined by a temperature of 39°C or higher, purulent nasal discharge, or facial pain, for at least 3 consecutive days; worsening course after initial improvement; or persistent illness (symptoms lasting ≥10 days).^16,17,18^ Among patients with sinusitis and suppurative OM,^15^ we examined antibiotic prescribing and its relationship to the timing of symptom onset and self-reported fever, when available.

### Statistical Analysis

Categorical data were analyzed with a χ^2^ test. We used logistic regression to develop 2 models with predictors of antibiotic prescribing. A model examining antibiotic prescribing among those with only tier 3 diagnoses adjusted for sex, site, age group, race, number of chronic conditions, time from symptom onset to presentation for care, and clinical diagnosis. A model that examined antibiotic prescribing among those with laboratory-confirmed influenza adjusted for sex, site, age group, number of chronic medical conditions, time from symptom onset, clinical diagnosis, and presence of fever. Variables with 2-sided *P* < .20 on univariate analysis were included in multivariable analyses and subjected to model-fitting procedures. A 2-sided *P* value less than .05 was considered statistically significant. Statistical analyses were conducted using SAS version 9.4 statistical software (SAS Institute Inc).

## Results

During the 2013-2014 and 2014-2015 influenza seasons, we enrolled 15 714 patients. Among these, 727 were excluded: 126 had missing *ICD-9* codes, 118 were prescribed an antibiotic while hospitalized, and 483 were diagnosed with a nonrespiratory condition for which antibiotics are potentially indicated. Among the 14 987 patients included, the mean (SD) age was 32 (24) years, 8638 (58%) were women, and 11 892 (80%) were white (Table 1). The 14 987 included patient visits had a mean (SD) of 2.0 (1.2) *ICD-9* codes; 338 (2%) had at least 5 codes, less than 1% of which had additional codes indicating tier 1 or tier 2 diagnoses. Results were unchanged when we analyzed all diagnosis codes.

**Table 1.** Characteristics of Patients With Acute Respiratory Infections in the 2013-2014 and 2014-2015 Influenza Seasons at Ambulatory Care Settings Affiliated With the US Influenza Vaccine Effectiveness Network^a^

Characteristic	Patients, No. (%)	
	Total (N = 14 987)^b^	Prescribed Antibiotic (n = 6136)^c^
Sex		
Male	6349 (42)	2567 (40)
Female	8638 (58)	3569 (41)
Age group, y		
0.5-2	918 (6)	398 (43)
2 to <5	1488 (10)	589 (40)
5 to <18	3294 (22)	1186 (36)
18 to <50	4948 (33)	1938 (39)
50 to <65	2563 (17)	1183 (46)
≥65	1776 (12)	842 (47)
Race		
White	11 892 (80)	5150 (43)
Black	1407 (9)	424 (30)
Other	1619 (11)	534 (33)
Study site		
A	4286 (29)	1353 (32)
B	3112 (21)	1629 (52)
C	2699 (18)	1166 (43)
D	2315 (15)	858 (37)
E	2575 (17)	1130 (44)
Time from symptom onset to presentation for care, d		
≤2	4678 (31)	1610 (34)
3-4	5719 (38)	2406 (42)
5-7	4590 (31)	2120 (46)
≥1 Chronic medical condition	5331 (36)	2315 (43)
Laboratory-confirmed influenza^d^	3381 (22)	1017 (30)

^a^ Acute respiratory infection is defined by cough with 7 days’ duration or less.

^b^ Percentage indicates the percentage in the column.

^c^ Percentage indicates the percentage in the row.

^d^ Tested by real-time reverse transcriptase–polymerase chain reaction in all enrolled patients. Testing was performed for research purposes. Clinicians were unaware of results, except for 1 site at which clinicians were notified of positive real-time reverse transcriptase–polymerase chain reaction research results within 48 hours of enrollment.

**Figure 1.**
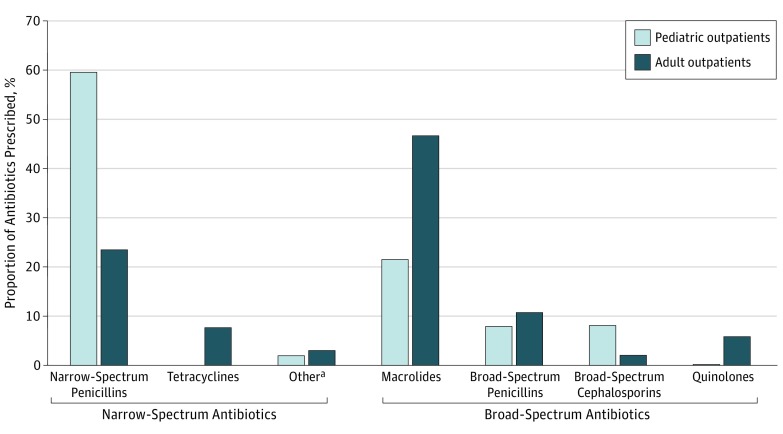
Antibiotic Classes Prescribed in Pediatric and Adult Outpatients With Acute Respiratory Infections in the 2013-2014 and 2014-2015 Influenza Seasons in the US Influenza Vaccine Effectiveness Network Acute respiratory infections are defined by cough with 7 days’ duration or less. Pediatric indicates patients aged 6 months to younger than 18 years. ^a^Includes first-generation cephalosporins and sulfonamides.

Among 14 987 patient visits analyzed, 6136 (41%) were associated with an antibiotic prescription, including 3423 (56%) with broad-spectrum antibiotics. Five drugs accounted for 90% of antibiotic prescriptions: azithromycin (37%), amoxicillin (35%), amoxicillin-clavulanate (10%), doxycycline (5%), and levofloxacin (3%) (Figure 1). Macrolides were most frequently prescribed in adults; 960 of 4339 adults (22%) aged 50 years or older were prescribed azithromycin, regardless of clinical diagnosis, symptoms, symptom duration, or laboratory findings.

### Influenza and Antibiotic Prescribing

**Figure 2.**
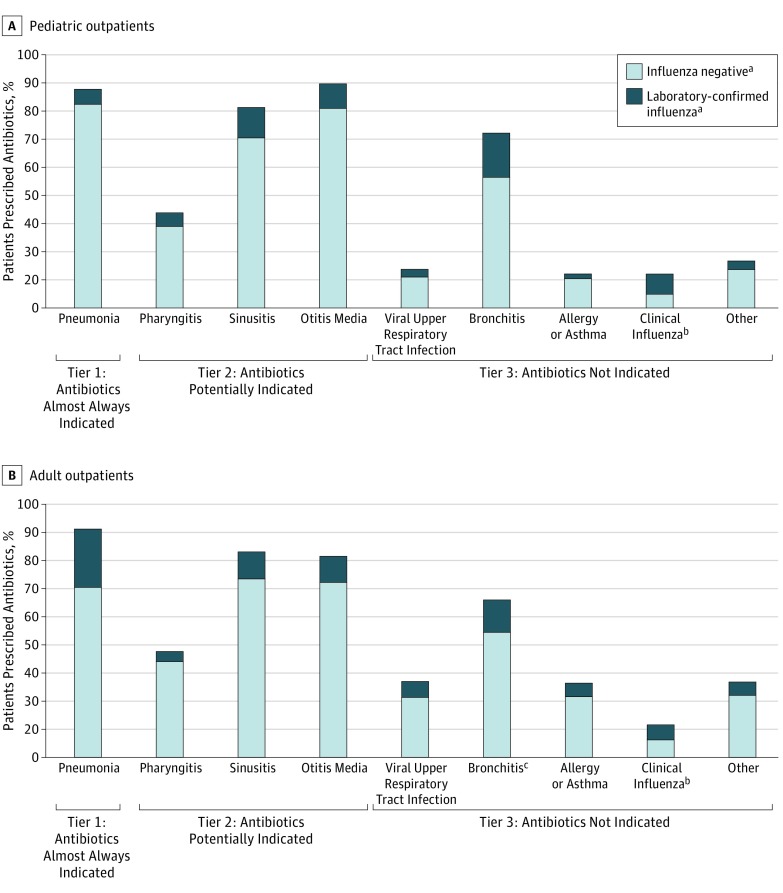
Proportion of Pediatric and Adult Outpatients With Acute Respiratory Infections Who Were Prescribed Antibiotics, by Selected *International Classification of Diseases, Ninth Revision *Diagnostic Codes and Laboratory-Confirmed Influenza Status Acute respiratory infections are defined by cough with 7 days’ duration or less. Pediatric indicates patients aged 6 months to younger than 18 years. ^a^All enrollees received influenza testing by real-time reverse transcriptase–polymerase chain reaction for research purposes only. At 1 site, clinicians were provided study real-time reverse transcriptase–polymerase chain reaction results for influenza within 48 hours of enrollment. Results were not available to clinicians at other sites. ^b^Indicates an *International Classification of Diseases, Ninth Revision* diagnosis code of influenza assigned by the clinician who saw the patient. ^c^Adults with a history of chronic obstructive pulmonary disease or a visit diagnosis code for chronic obstructive pulmonary disease were excluded from the analysis of bronchitis.

Influenza was confirmed through research testing in 3381 patients (23%), 902 (27%) of whom received a clinical diagnosis code for influenza. Excluding those with a tier 1 diagnosis, among 3306 patients (22%) with laboratory-confirmed influenza, 945 (29%) were prescribed an antibiotic, accounting for 17% of all antibiotic prescriptions among patients with nonpneumonia ARI; 656 (20%) were prescribed an influenza antiviral medication, including 89 prescribed both antibiotic and antiviral medications. Laboratory-confirmed influenza was detected in 72 of 375 patients (16%) diagnosed as having pneumonia, 446 of 2065 patients (18%) with pharyngitis, 129 of 1099 patients (11%) with suppurative OM, and 227 of 1486 patients (13%) with sinusitis; the proportion with laboratory-confirmed influenza among those prescribed antibiotics is shown in Figure 2. In a multivariable model among those with laboratory-confirmed influenza, antibiotic prescribing was significantly associated with a clinical diagnosis of pneumonia (adjusted odds ratio [AOR] = 145.74; 95% CI, 34.37-617.82), sinusitis (AOR = 15.34; 95% CI, 9.83-23.94), OM (AOR = 71.77; 95% CI, 30.00-171.71), and bronchitis (AOR = 4.60; 95% CI, 3.27-6.47), but not pharyngitis (AOR = 1.23; 95% CI, 0.90-1.69). Other factors associated with antibiotic prescribing include site, longer symptom duration, and older age. Those with a clinical diagnosis code for influenza (AOR = 0.30; 95% CI, 0.21-0.41), URI (AOR = 0.75; 95% CI, 0.57-0.99), or other tier 3 diagnosis (AOR = 0.50; 95% CI, 0.33-0.76) had decreased odds of receiving an antibiotic prescription (eFigure 1 in the Supplement). At 4 sites, clinical nonresearch influenza testing was performed on the day of enrollment for 198 of 12 412 patients (2%).

### Antibiotic Prescribing Without an Appropriate Indication (Tier 3 Diagnoses)

Among 6136 patients who received antibiotic prescriptions, 2522 (41%) had only diagnoses for which antibiotics are not indicated (tier 3) (Table 2; eFigure 2 in the Supplement). The proportion of patients with only tier 3 diagnoses who were prescribed antibiotics increased with increasing age: 25%, 46%, and 65% of antibiotic prescriptions among those younger than 18 years, 18 to 64 years, and 65 years or older, respectively (*P* < .01). Azithromycin was the most commonly prescribed antibiotic given to adults who lacked a diagnosis for which antibiotics are indicated; among the 3186 adults aged 50 years and older with no clinical indication for antibiotics (tier 3 diagnoses only), 1183 (37%) received an antibiotic prescription; 688 (58%) of these prescriptions were for azithromycin.

**Table 2.** Antibiotic Prescribing by Diagnostic Category Tier Among Patients With Acute Respiratory Infections in the 2013-2014 and 2014-2015 Influenza Seasons at Ambulatory Care Settings Affiliated With the US Influenza Vaccine Effectiveness Network^a^

Diagnosis	Patients, No. (%)					
	All Ages		Pediatric		Adult	
	Total (N = 14 987)^b^	Prescribed Antibiotic (n = 6136)^c^	Total (n = 5700)^b^	Prescribed Antibiotic (n = 2173)^c^	Total (n = 9287)^b^	Prescribed Antibiotic (n = 3963)^c^
Tier 1: antibiotics almost always indicated	467 (3)	416 (89)	218 (4)	187 (86)	249 (3)	229 (92)
Pneumonia	447 (3)	400 (89)	202 (4)	174 (86)	245 (3)	226 (92)
Miscellaneous bacterial infections	20 (0.1)	16 (80)	16 (0.3)	13 (81)	4 (0.04)	3 (75)
Tier 2: antibiotics potentially indicated^d^	5129 (34)	3198 (62)	2322 (41)	1445 (62)	2807 (30)	1753 (62)
Pharyngitis	2494 (17)	1000 (40)	1167 (20)	439 (38)	1327 (14)	561 (42)
Sinusitis	1707 (11)	1385 (81)	243 (4)	192 (79)	1464 (16)	1193 (81)
Suppurative otitis media	1212 (8)	1055 (87)	993 (17)	881 (89)	219 (2)	174 (79)
Tier 3: antibiotics not indicated^e^	9391 (63)	2522 (27)	3160 (55)	541 (17)	6231 (67)	1981 (32)
Viral upper respiratory tract infection	5553 (37)	1376 (25)	1964 (34)	299 (15)	3589 (39)	1077 (30)
Bronchitis^f^	1157 (8)	730 (63)	107 (2)	74 (7)	1050 (11)	656 (62)
Allergy or asthma	1217 (8)	289 (24)	451 (8)	61 (14)	766 (8)	228 (30)
Influenza diagnosis code^g^	1257 (8)	167 (13)	317 (6)	43 (14)	940 (10)	124 (13)
Viral pneumonia	3 (0.02)	2 (67)	2 (0.04)	1 (50)	1 (0.01)	0
Remaining codes not listed elsewhere	1463 (10)	373 (25)	602 (11)	113 (19)	861 (9)	260 (30)

^a^ The diagnostic category tiers are adapted from Fleming-Dutra et al^2^ and Shapiro et al.^14^ The first 4 *International Classification of Diseases, Ninth Revision* diagnostic codes were examined. Each patient was classified in a tier; priority was given to tier 1 diagnoses, then tier 2 diagnoses, then tier 3 diagnoses. However, individuals could be in more than 1 diagnosis category.

^b^ Percentage indicates the percentage in the column.

^c^ Percentage indicates the percentage in the row.

^d^ Excludes patients with any tier 1 diagnosis.

^e^ Excludes patients with any tier 1 or tier 2 diagnosis.

^f^ Excludes 31 adults with a history of chronic obstructive pulmonary disease per medical record or who had a chronic obstructive pulmonary disease diagnosis code. If these are included, 684 of 1081 adults (63%) with a diagnosis of bronchitis received antibiotics.

^g^ Indicates an *International Classification of Diseases, Ninth Revision* code diagnosis of influenza, not laboratory confirmation of influenza, which was performed for research purposes only.

**Figure 3.**
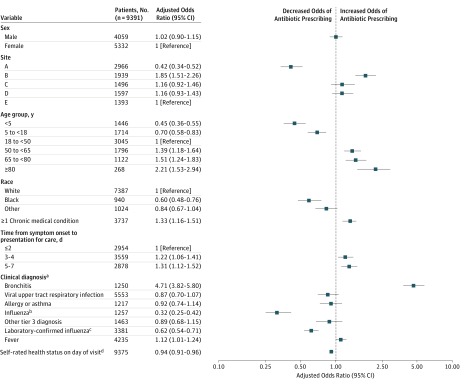
Multivariable Analysis of Predictors of Antibiotic Prescribing Among Persons With Acute Respiratory Infections and Assigned Diagnosis Codes for Which Antibiotics Are Not Indicated (Tier 3 Diagnoses Only) ^a^By *International Classification of Diseases, Ninth Revision* diagnosis code. ^b^Indicates an *International Classification of Diseases, Ninth Revision* code diagnosis of influenza, not real-time reverse transcriptase–polymerase chain reaction confirmation of influenza. ^c^Indicates real-time reverse transcriptase–polymerase chain reaction confirmation of influenza, which was performed for research purposes only. ^d^Self-rated health on a scale of 0 (worst) to 100 (best), analyzed as a continuous variable.

Among the 2522 patients with tier 3 diagnoses prescribed an antibiotic, 2106 (84%) were diagnosed as having either a viral URI or bronchitis (acute or not otherwise specified). Among 5553 patients with a viral URI diagnosis, 1376 (25%) received an antibiotic, accounting for 22% of all antibiotic prescriptions (Figure 2). Excluding patients with COPD, 1157 persons were assigned a diagnosis for bronchitis; 730 (63%) received an antibiotic, accounting for 12% of all antibiotic prescriptions. In a multivariable model among those with only tier 3 diagnoses, antibiotic prescribing was most strongly associated with a diagnosis of bronchitis (AOR = 4.71; 95% CI, 3.82-5.80) (Figure 3). Other factors associated with antibiotic prescribing include site, longer symptom duration, older age, at least 1 chronic medical condition, worse self-rated health, and fever. Black patients were significantly less likely to be prescribed antibiotics than white patients (AOR= 0.60; 95% CI, 0.48-0.76; *P* < .001). Both a clinical diagnosis of influenza and laboratory-confirmed influenza through research testing were significantly associated with decreased odds of an antibiotic prescription (clinical diagnosis: AOR = 0.32; 95% CI, 0.25-0.42; laboratory-confirmed diagnosis: AOR = 0.62; 0.54-0.71).

### Pharyngitis, Sinusitis, and OM (Tier 2 Diagnoses)

The 3 most frequent diagnoses for which antibiotics are potentially indicated (tier 2 diagnoses) were pharyngitis (diagnosed in 2494 patients [17%]), sinusitis (1707 patients [11%]), and suppurative OM (1212 patients [8%]), which together accounted for 52% of all antibiotics prescribed. Among 3198 persons who received antibiotics for these diagnoses, 2050 (64%) received first-line antibiotics (eFigure 2 and eTable 2 in the Supplement).

Pharyngitis accounted for 16% of antibiotic prescriptions. Among 1000 of 2494 patients (40%) with a pharyngitis diagnosis who received an antibiotic, 122 (12%) had laboratory-confirmed influenza (Figure 2; eTable 2 in the Supplement). Among the 1248 patients assigned a pharyngitis diagnosis code who had information on GAS testing available, 1137 (91%) had GAS testing performed. A total of 440 patients with pharyngitis (35%) were prescribed an antibiotic, among whom 208 (47%) lacked a positive GAS test, either because they did not have testing performed (40 patients [9%]) or their test result was negative (168 patients [38%]) (eFigure 3 in the Supplement). An estimated 327 of 860 patients (38%) in the entire study population with only a pharyngitis diagnosis were given an antibiotic prescription despite a negative GAS test result.

Among 1707 patients assigned a sinusitis diagnosis, 1385 (81%) were prescribed antibiotics, 188 (14%) of whom had laboratory-confirmed influenza (Figure 2; eTable 2 in the Supplement). Among the 1295 patients diagnosed as having sinusitis for whom fever information was collected, 1087 (84%) were prescribed an antibiotic; 558 (51%) reported a fever, whereas 203 (19%) had neither fever nor symptom duration longer than 3 days. Among patients with sinusitis and no other indication for antibiotic treatment who received an antibiotic, the median time from symptom onset to prescription date was 4 days (interquartile range, 3-6 days); 454 of 1200 patients (38%) were prescribed an antibiotic within 3 days following symptom onset, a proportion that was unchanged when these patients were stratified by fever status.

Suppurative OM accounted for 17% of antibiotic prescriptions, including 41% of those in children and 4% in adults. Among 1055 persons with this diagnosis prescribed an antibiotic, 121 (11%) had laboratory-confirmed influenza (Figure 2; eTable 2 in the Supplement).

Among all 6136 patients with antibiotic prescriptions, we estimated 3303 (54%) likely did not meet clinical criteria or laboratory criteria for GAS pharyngitis for antibiotic treatment. Of these 6136 patients, 2522 (41%) had only clinical diagnoses that do not warrant antibiotics (tier 3 diagnoses only), 454 (7%) had sinusitis that did not meet clinical criteria, and an estimated 327 (5%) had pharyngitis with a negative GAS test result. Among the remaining 2833 patients given antibiotic prescriptions who had a clinical diagnosis for which antibiotic therapy may be indicated (eg, suppurative OM, pharyngitis, or sinusitis), an additional 369 (13%) had influenza virus infection confirmed through research testing and likely did not benefit from antibiotic therapy.

## Discussion

Antibiotics were likely prescribed inappropriately to a majority of the nearly 15 000 outpatients in this study who presented during influenza season with symptoms of a broadly defined ARI characterized by cough. Among all patients prescribed antibiotics, 41% lacked a diagnosis code for which antibiotic therapy is potentially indicated. Those with influenza confirmed through research testing accounted for a substantial proportion (17%) of all antibiotics prescribed; fewer than one-third of patients with laboratory-confirmed influenza were given a clinical diagnosis of influenza. Patients with influenza virus infection accounted for a substantial proportion of those given antibiotics for diagnoses for which antibiotics may be appropriate, including pharyngitis (12%), sinusitis (14%), and suppurative OM (11%), although most patients with influenza are unlikely to benefit from antibiotic treatment. In addition, among those patients given diagnoses for which antibiotic therapy may be appropriate, many patients prescribed antibiotics likely did not meet criteria for antibiotic therapy based on clinician-ordered laboratory testing and clinical criteria, including 47% of those with a pharyngitis diagnosis and 38% with a sinusitis diagnosis.

Our study is consistent with previous studies that indicate clinicians overprescribe antibiotics for outpatient ARIs. These studies used national survey data, which lack detailed clinical data, including laboratory testing results.^2,4,5,6,7^ We used a sensitive clinical definition of ARI and had data including illness onset date, laboratory confirmation of influenza, and, for many patients, self-reported fever and the results of clinician-ordered GAS testing. As noted in previous studies,^2,6^ we found that a substantial proportion of antibiotic overuse was driven by prescribing for conditions for which antibiotics were not indicated, including viral URIs and acute bronchitis.^19^ Our results also suggest antibiotics were frequently overprescribed for patients with 2 common diagnoses for which antibiotics are potentially indicated, pharyngitis and sinusitis, underscoring that these diagnoses are important targets for improving antibiotic use. For pharyngitis, national guidelines recommend antibiotic therapy only for laboratory-confirmed GAS^19,20^; in our study, nearly half of patients diagnosed as having pharyngitis who received antibiotics either tested negative for GAS or were not tested. For patients with sinusitis, among those prescribed an antibiotic, 38% had symptoms for 3 days or less and most did not report fever, indicating that many patients likely did not meet clinical criteria for antibiotic treatment as defined by national guidelines.

Older adults were more likely than younger adults and children to receive antibiotics without diagnostic documentation of an appropriate indication and were far more likely to receive broad-spectrum antibiotics, particularly macrolides. Azithromycin was prescribed to nearly one-quarter of adults aged 50 years or older with an ARI, regardless of diagnosis, laboratory testing, symptom duration, or medical history, including to 58% of those older adults given a prescription who lacked an indication for antibiotic treatment. Azithromycin also accounted for more than one-quarter of prescriptions among those adults diagnosed as having pharyngitis and sinusitis, even though it is not the first-line agent for either condition. A concerning increase in macrolide use for these conditions has been previously noted in both adults and children.^5,7^ Choosing a macrolide when amoxicillin or amoxicillin-clavulanate is the recommended first-line antibiotic is a potential patient safety issue; GAS and *Streptococcus pneumoniae* infections are more likely to be resistant to macrolides than to amoxicillin or amoxicillin-clavulanate. While all antibiotics have potential risks, macrolides have also been associated with an increased risk of cardiovascular events.^21^

This study was conducted during influenza seasons, and we found that 17% of patients with ARI prescribed antibiotics had laboratory-confirmed influenza. Influenza can predispose patients to increased risk of bacterial coinfections. In some instances of influenza infection, antibiotics may be appropriately prescribed if an associated bacterial infection is suspected. However, bacterial coinfection was likely only in a minority of influenza cases.^22^ As in previous studies,^10^ we found that patients with influenza confirmed through research testing were more likely to receive an antibiotic than an antiviral medication. In addition, a substantial proportion of patients with diagnoses for which antibiotics may be considered appropriate—pharyngitis, suppurative OM, and sinusitis—actually had influenza virus infection, and depending on the clinical situation, many would likely not benefit from antibiotic treatment. Current widely available point-of-care influenza diagnostic tests have highly variable sensitivity^8^ and are not recommended for ruling out influenza infection for the purposes of treatment decisions^9^; clinician-ordered testing was infrequent in this study. Clinicians assigned an influenza diagnosis code to only one-quarter of patients with influenza confirmed through research testing, indicating that influenza virus infections were underdiagnosed. However, among all patients with ARI, those patients to whom clinicians assigned an influenza diagnosis were significantly less likely to receive an antibiotic prescription. The development of sensitive and specific point-of-care testing for influenza may assist clinicians in making treatment decisions for patients with ARI during influenza season and may help to reduce unnecessary antibiotic use for influenza in outpatient settings.^23^ Inappropriate antibiotic prescribing exposes patients to the risks of unnecessary antibiotics and represents a potential missed opportunity for patients to benefit from influenza antiviral medications.^9^ Clinicians should be encouraged to consider influenza as a clinical diagnosis during the influenza season, refrain from prescribing antibiotics in situations in which they are not recommended by guidelines, and prescribe influenza antiviral medications when indicated.^9^

### Limitations

This study has several limitations. We used up to 4 *ICD-9* codes to determine whether antibiotics were prescribed appropriately, but these may inaccurately reflect clinical decision making. Medical history information may have been incomplete in some instances, and other factors may have informed prescribing decisions. Our study sites may not be representative of other outpatient settings; some sites are health care organizations that may have institutional prescribing policies or mostly serve insured patients. In addition, sites have been engaged in influenza research for multiple years, which may have influenced clinician awareness of influenza and antibiotic prescribing practices. Of note, the proportion of patients prescribed antibiotics for conditions such as pharyngitis and bronchitis was lower in this study compared with other studies, and assessment of antibiotic overuse may be underestimated.^2,6,7^ We also assumed prescriptions were related to the study visit, but this could not always be verified. We lacked dispensing information for several sites, limiting our ability to know whether clinicians used delayed prescribing strategies in which patients are instructed to fill prescriptions only if symptoms failed to improve. This could have led to an overestimation of antibiotic use. Previous antibiotic treatment of prior infections and allergy history were not available, both of which may have affected antibiotic choice.

## Conclusions

Our study adds to evidence that misuse of antibiotics, characterized by antibiotic overuse and inappropriate antibiotic selection, is widespread in the treatment of outpatient ARIs. The study indicates a number of potential targets to achieve the goal of the National Action Plan for Combating Antibiotic-Resistant Bacteria of reducing inappropriate outpatient antibiotic use by 50% by 2020.^24^ We must strengthen outpatient antibiotic stewardship efforts to eliminate antibiotic treatment for viral URIs and acute bronchitis, which our study indicates would make the largest contribution to decreasing unnecessary antibiotic prescriptions. Increased efforts are needed to support improved adherence to guidelines for antibiotic prescribing for common diagnoses, including more stringent adherence to GAS pharyngitis testing guidelines and clinical criteria for antibiotic treatment of sinusitis, as well as interventions focused on appropriate selection of first-line antibiotics for these conditions if treatment is indicated. In addition, our findings indicate that improved point-of-care influenza diagnostics and increased recognition and appropriate treatment of influenza virus infection may also aid in decreasing unnecessary antibiotic use for ARIs. The Centers for Disease Control and Prevention published “Core Elements of Outpatient Antibiotic Stewardship,”^25^ which provides guidance to clinicians and facility leadership to implement activities to improve antibiotic use. Improving antibiotic prescribing for ARIs during the influenza season represents an important opportunity to improve the long-term quality of patient care.

## Notes

Supplement. **eTable 1.** Diagnostic Categories by Tier With Corresponding *International Classification of Diseases, Ninth Revision* (*ICD-9*) Diagnosis Codes **eTable 2.** Antibiotic Selection Among Those With an *International Classification of Diseases, Ninth Revision* (*ICD-9*) Diagnosis Code for Pharyngitis, Sinusitis, and Suppurative Otitis Media Among Those Prescribed Antibiotics **eFigure 1.** Predictors of Antibiotic Prescribing Among Persons With Laboratory-Confirmed Influenza and Who Sought Care at Ambulatory Care Settings Associated With 5 Institutions, US Influenza Vaccine Effectiveness Network, 2013-2014 and 2014-2015 Influenza Seasons **eFigure 2.** Number of Enrollees With Acute Respiratory Infections Defined by Cough ≤7 Days’ Duration, Selected *ICD-9* Diagnostic Codes, by Antibiotic Prescribing Status **eFigure 3.** Antibiotic Prescribing Among Those With an *International Classification of Diseases, Ninth Revision* (*ICD-9*) Diagnosis Code for Pharyngitis, by Group A Streptococcus Testing Status, 2014-2015 Influenza Season
