# Supplementary material for: Outpatient Antibiotic Prescribing for Acute Respiratory Infections During Influenza Seasons
Source: JAMA Netw Open. 2018 Jun 8;1(2):e180243. doi: 10.1001/jamanetworkopen.2018.0243 (PMC6324415; doi:10.1001/jamanetworkopen.2018.0243)
Supplement: Supplement. — eTable 1. Diagnostic Categories by Tier With Corresponding International Classification of Diseases, Ninth Revision (ICD-9) Diagnosis Codes eTable 2. Antibiotic Selection Among Those With an International Classification of Diseases, Ninth Revision (ICD-9) Diagnosis Code for Pharyngitis, Sinusitis, and Suppurative Otitis Media Among Those Prescribed Antibiotics eFigure 1. Predictors of Antibiotic Prescribing Among Persons With Laboratory-Confirmed Influenza and Who Sought Care at Ambulatory Care Settings Associated With 5 Institutions, US Influenza Vaccine Effectiveness Network, 2013-2014 and 2014-2015 Influenza Seasons eFigure 2. Number of Enrollees With Acute Respiratory Infections Defined by Cough ≤7 Days’ Duration, Selected ICD-9 Diagnostic Codes, by Antibiotic Prescribing Status eFigure 3. Antibiotic Prescribing Among Those With an International Classification of Diseases, Ninth Revision (ICD-9) Diagnosis Code for Pharyngitis, by Group A Streptococcus Testing Status, 2014-2015 Influenza Season [file jamanetwopen-1-e180243-s001.pdf]

Supplementary Online Content

Havers FP, Hicks LA, Chung JR, et al. Outpatient antibiotic prescribing for acute respiratory infections during influenza seasons. *JAMA Netw Open*. 2018;1(2):e180243.
doi:10.1001/jamanetworkopen.2018.0243

eTable 1. Diagnostic Categories by Tier With Corresponding *International Classification of Diseases, Ninth Revision* (*ICD-9*) Diagnosis Codes

eTable 2. Antibiotic Selection Among Those With an *International Classification of Diseases, Ninth Revision* (*ICD-9*) Diagnosis Code for Pharyngitis, Sinusitis, and Suppurative Otitis Media Among Those Prescribed Antibiotics

eFigure 1. Predictors of Antibiotic Prescribing Among Persons With Laboratory-Confirmed Influenza and Who Sought Care at Ambulatory Care Settings Associated With 5 Institutions, US Influenza Vaccine Effectiveness Network, 2013-2014 and 2014-2015 Influenza Seasons

eFigure 2. Number of Enrollees With Acute Respiratory Infections Defined by Cough ≤7 Days' Duration, Selected *ICD-9* Diagnostic Codes, by Antibiotic Prescribing Status

eFigure 3. Antibiotic Prescribing Among Those With an *International Classification of Diseases, Ninth Revision* (*ICD-9*) Diagnosis Code for Pharyngitis, by Group A Streptococcus Testing Status, 2014-2015 Influenza Season

This supplementary material has been provided by the authors to give readers additional information about their work.

© 2018 Havers FP et al. *JAMA Network Open.*

eTable 1. Diagnostic categories by tier[a] with corresponding *International Classification of Diseases, Ninth Revision* (*ICD-9*) diagnosis codes. Adapted from Fleming-Dutra, et al.,[2] and Shapiro, et al.[14]

**Diagnosis**	**Corresponding ICD-9CM codes**
**Tier 1 diagnoses: Antibiotics almost always indicated**	
Pneumonia	481 Pneumococcal pneumonia [*Streptococcus pneumoniae* pneumonia], 482 Other bacterial pneumonia, 483 Pneumonia due to other specified organism, 484 Pneumonia in infectious diseases classified elsewhere, 485 Bronchopneumonia, organism unspecified, 486 Pneumonia, organism unspecified
Miscellaneous bacterial infections	010-018, 020-027, 030-033, 036- 041,080-104, 130-139, 320-323, 383, 475<br>Various named infections including tuberculosis, zoonotic bacterial diseases, diphtheria, pertussis, rickettsial and venereal diseases, meningitis, mastoiditis,
Urinary tract infections (UTI) (excluded)[b]	590.1, 590.2, 590.8, 590.9, 595.0, 595.9, 599.0: Acute pyelonephritis, renal abscess, other pyelonephritis/pyelonephrosis, kidney infection (unspecified), acute cystitis, cystitis (unspecified), UTI (unspecified)
**Tier 2 diagnoses: Antibiotics may be indicated**	
Pharyngitis	462 Acute pharyngitis, 463 Acute tonsillitis, 034 Streptococcal sore throat and scarlet fever
Sinusitis	461 Acute sinusitis, 473 Chronic sinusitis
Suppurative otitis media	382 Suppurative and unspecified otitis media
Acne (excluded)[b]	706.0, 706.1 Acne, including vulgaris and varioloformis
Gastrointestinal infections (excluded)[b]	001-009, 787, 789 Intestinal infectious diseases, symptoms involving the abdomen or digestive system
Skin, cutaneous and mucosal infections (excluded)[b]	680-686, 035, 110-111, 704.8, 728.0, 611.0, 771.5, 728.86, 380.0-380.1 Includes cellulitis, carbuncle/furuncle, impetigo, lymphadenitis, erysipelas, dermatophytosis, folliculitis, myositis, mastitis, necrotizing fasciitis, infective otitis externa
**Tier 3 diagnoses: Antibiotics not indicated**	
Asthma, allergy	493 Asthma, 477 Allergic rhinitis, 995.3 Allergy, unspecified
Bronchitis, bronchiolitis	490 Bronchitis, not specified as acute or chronic, 466.0 Acute bronchitis, 466.1 bronchiolitis, **Excludes visits which included a diagnosis of chronic bronchitis (491), emphysema (492), or COPD (496) or patients who had a one of these diagnosis codes within the preceding 12 months.
Influenza	487 Influenza, 488 Influenza due to identified avian influenza virus
Miscellaneous other infections	042, 045-049, 050-059, 070-079, 112-129: Human immunodeficiency virus (HIV) infection, poliomyelitis and other non-arthropod-borne viral diseases and prion disease of central nervous system, viral hepatitis, mumps, coxsackie virus,viral disease accompanied by exanthema, mycoses, helminthiases
Non- suppurative otitis media	381: Non-suppurative otitis media and Eustachian tube disorders
Other gastrointestinal conditions	520-579 Other conditions of the digestive system, not already included under gastrointestinal infections
Other skin, cutaneous and mucosal conditions	690-698, 700-709, 870-897, 910-919, 940-949, 360-379, 380-389 (excel 380.0-380.1), 782, 785.4, 785.6: Skin, cutaneous and mucosal conditions not already categorized as skin, cutaneous and mucosal infections: includes inflammatory and other skin conditions, open wounds, superficial injuries, burns, diseases of the eye/adnexa, ear diseases other than otitis media and mastoiditis, symptoms of skin/integumentary tissues (excluding acne), gangrene, enlargement of lymph nodes

© 2018 Havers FP et al. *JAMA Network Open.*

Other genitourinary conditions	580-629, 788.1 (excluding UTI codes above): Other genitourinary codes not already categorized as UTI, Other conditions of the genitourinary system, symptoms involving urinary system (including dysuria)
Viral pneumonia	480: Viral pneumonia
Viral upper respiratory infection (URI)	460: Acute nasopharyngitis [common cold], 464 Acute laryngitis and tracheitis, 465 Acute upper respiratory infections of multiple or unspecified sites, 786.2 Cough
Other respiratory conditions	All remaining respiratory conditions (460-519) not coded above and 786.0-786.1, 786.3-786.4: Includes chronic bronchitis and bronchitis with a 2nd or 3rd diagnosis of chronic bronchitis, emphysema and COPD; other respiratory conditions; dyspnea; stridor; hemoptysis; abnormal sputum
All other codes not listed elsewhere	All remaining codes not listed elsewhere

[a] In assigning each visit a single diagnosis, priority was given to Tier 1 diagnoses, then Tier 2 diagnoses, then Tier 3 diagnoses.

[b] Patients with ICD-9 codes for non-respiratory conditions for which antibiotics are almost always indicated (Tier 1) or may be indicated (Tier 2) were excluded from the analysis.

© 2018 Havers FP et al. *JAMA Network Open.*

eTable 2. Antibiotic selection[a] among those with an International Classification of Diseases, Ninth Revision (ICD-9) diagnosis code for pharyngitis, sinusitis and suppurative otitis media among those prescribed antibiotics.

	All ages n (%)	Adult n (%)	Pediatrics n (%)
**Pharyngitis**	1000	561	439
First-line antibiotics	593 (59)	276 (49)	317 (72)
Amoxicillin	496 (50)	205 (37)	291 (66)
Penicillin	97 (10)	71 (13)	26 (6)
Non first-line antibiotics	407 (41)	285 (51)	122 (28)
Amoxicillin-clavulanate	60 (6)	46 (8)	14 (3)
Macrolides	229 (23)	159 (28)	70 (16)
Fluoroquinolones	11 (1)	11 (2)	0 (0)
Broad-spectrum cephalosporins	23 (2)	9 (2)	14 (3)
Tetracycline antibiotics	20 (2)	20 (2)	0 (0)
Other	64 (6)	40 (6)	24 (5)
**Sinusitis**	1385	1193	192
First-line antibiotics	804 (58)	671 (58)	133 (69)
Amoxicillin	538 (39)	431 (36)	107 (56)
Amoxicillin-clavulanate	266 (19)	240 (20)	26 (14)
Non first-line antibiotics	581 (42)	522 (44)	59 (31)
Macrolides	365 (26)	325 (27)	40 (21)
Fluoroquinolone	41 (3)	41 (3)	0 (0)
Broad-spectrum cephalosporins	44 (3)	29 (2)	15 (8)
Tetracycline antibiotics	80 (6)	80 (7)	0 (0)
Other	51 (4)	47 (4)	4 (2)
**Suppurative otitis media**	1,055	174	881
First-line antibiotics	841 (80)	124 (71)	717 (81)
Amoxicillin	729 (69)	91 (52)	638 (72)
Amoxicillin-clavulanate	112 (11)	33 (19)	79 (9)
Non first-line antibiotics			
Macrolides	92 (9)	30 (17)	62 (7)
Broad-spectrum cephalosporins	98 (9)	4 (2)	94 (11)
Fluoroquinolones	5 (1)	4 (2)	1 (0)
Other	19 (7)	12 (2)	7 (1)

[a] Identification of first-line antibiotics as previously described: Hersh AL, Fleming-Dutra KE, Shapiro DJ, Hyun DY, Hicks LA, Outpatient Antibiotic Use Target-Setting W. Frequency of First-line Antibiotic Selection Among US Ambulatory Care Visits for Otitis Media, Sinusitis, and Pharyngitis. *JAMA internal medicine.* 2016.

© 2018 Havers FP et al. *JAMA Network Open.*

eFigure 1. Predictors of antibiotic prescribing among persons with laboratory-confirmed influenza and who sought care at ambulatory care settings associated with 5 institutions, US Influenza Vaccine Effectiveness Network, 2013-2014 and 2014-2015 influenza seasons.

		aOR[a]	95% CI
Male sex		1.05	(0.86 — 1.29)
Site	A	**0.28**	**(0.18 — 0.42)**
(Ref: E)	B	**1.55**	**(1.12 — 2.14)**
	C	0.76	(0.51 — 1.15)
	D	0.75	(0.53 — 1.05)
Age group	<5 years	0.79	(0.52 — 1.20)
(Ref: 18 to <50 years)	5 to <18 years	0.85	(0.64 — 1.13)
	50 to <65 years	**1.40**	**(1.04 — 1.88)**
	65 to <80 years	**1.87**	**(1.31 — 2.67)**
	≥80 years	**2.29**	**(1.27 — 4.11)**
Time from symptom onset	3-4 days	1.13	(0.90 — 1.43)
(Ref: ≤2 days)	5-7 days	**1.44**	**(1.09 — 1.90)**
≥1 medical condition		1.13	(0.90 — 1.41)
Clinical diagnosis (ICD9 code)	Pneumonia	**145.7**	**(34.4 — 617)**
	Sinusitis	**15.34**	**(9.83 — 23.94)**
	Pharyngitis	1.23	(0.90 — 1.69)
	Otitis media	**71.77**	**(30.0 — 171.7)**
	Upper respiratory infection	**0.75**	**(0.57 — 0.99)**
	Bronchitis	**4.60**	**(3.27 — 6.47)**
	Influenza code	**0.30**	**(0.21 — 0.41)**
	Other diagnosis	**0.50**	**(0.33 — 0.76)**
Fever		1.00	(0.82 — 1.21)

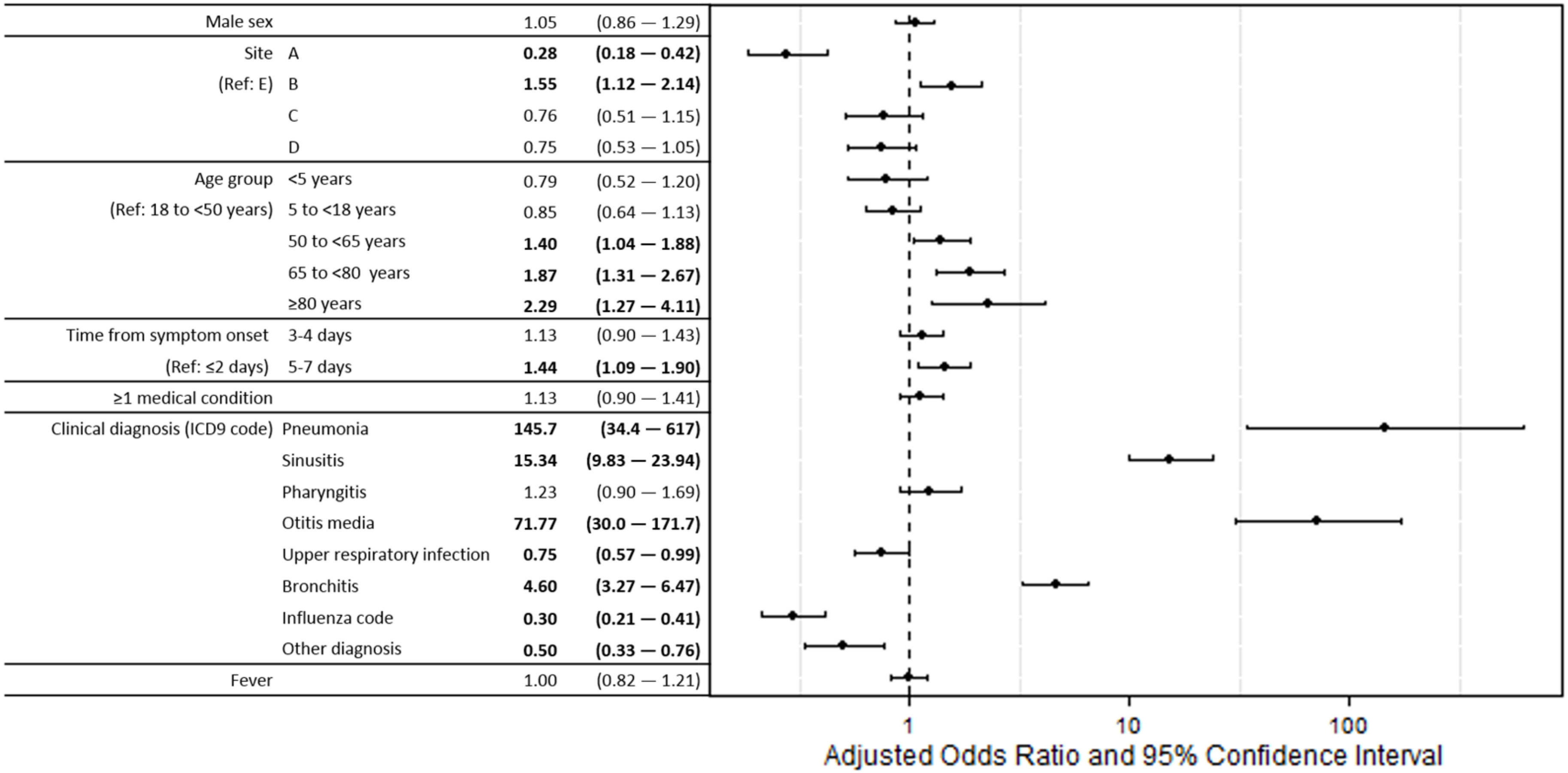

[a] aOR: Adjusted odds ratio. Ref: Reference value. Note: Influenza code: Indicates an International Classification of Diseases, Ninth Revision (ICD-9) code diagnosis of influenza, not reverse transcription polymerase chain reaction (RT-PCR) confirmation of influenza, which was performed on all enrollees for research purposes only.

© 2018 Havers FP et al. *JAMA Network Open.*

eFigure 2. Number of enrollees with acute respiratory infections defined by cough ≤7 days' duration, selected *ICD-9* diagnostic codes, by antibiotic prescribing status.

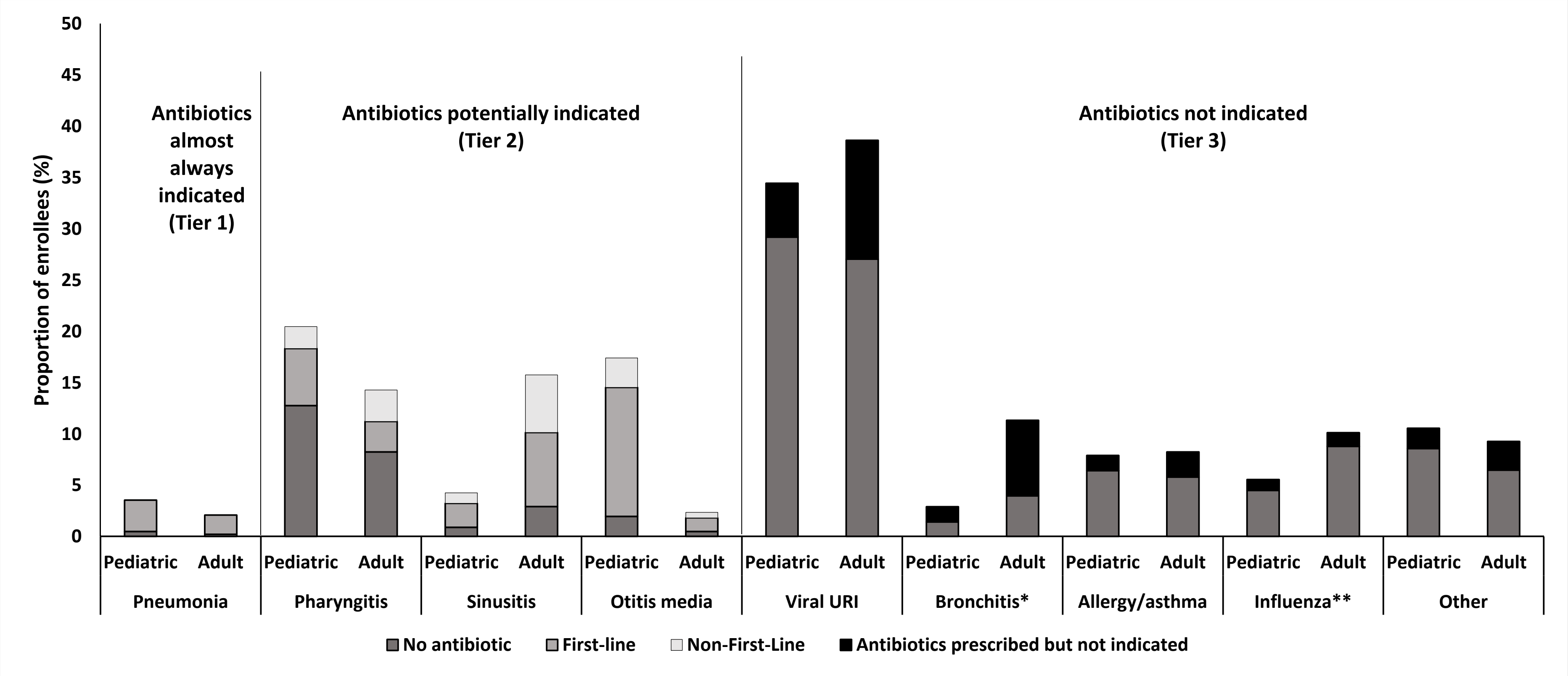

* Patients with a history of chronic obstructive pulmonary disease (COPD) or a visit diagnosis code for COPD were excluded from the analysis of bronchitis.

**Influenza: Indicates an ICD9 code diagnosis of influenza, not RT-PCR-confirmation of influenza which was performed for research purposes only.

Identification of first-line antibiotics as previously described: Hersh AL, Fleming-Dutra KE, Shapiro DJ, Hyun DY, Hicks LA, Outpatient Antibiotic Use Target-Setting W. Frequency of First-line Antibiotic Selection Among US Ambulatory Care Visits for Otitis Media, Sinusitis, and Pharyngitis. *JAMA internal medicine.* 2016.

© 2018 Havers FP et al. *JAMA Network Open.*

eFigure 3. Antibiotic prescribing among those with an *International Classification of Diseases, Ninth Revision* (*ICD-9*) diagnosis code for pharyngitis, by Group A streptococcus testing status, 2014-2015 influenza season.

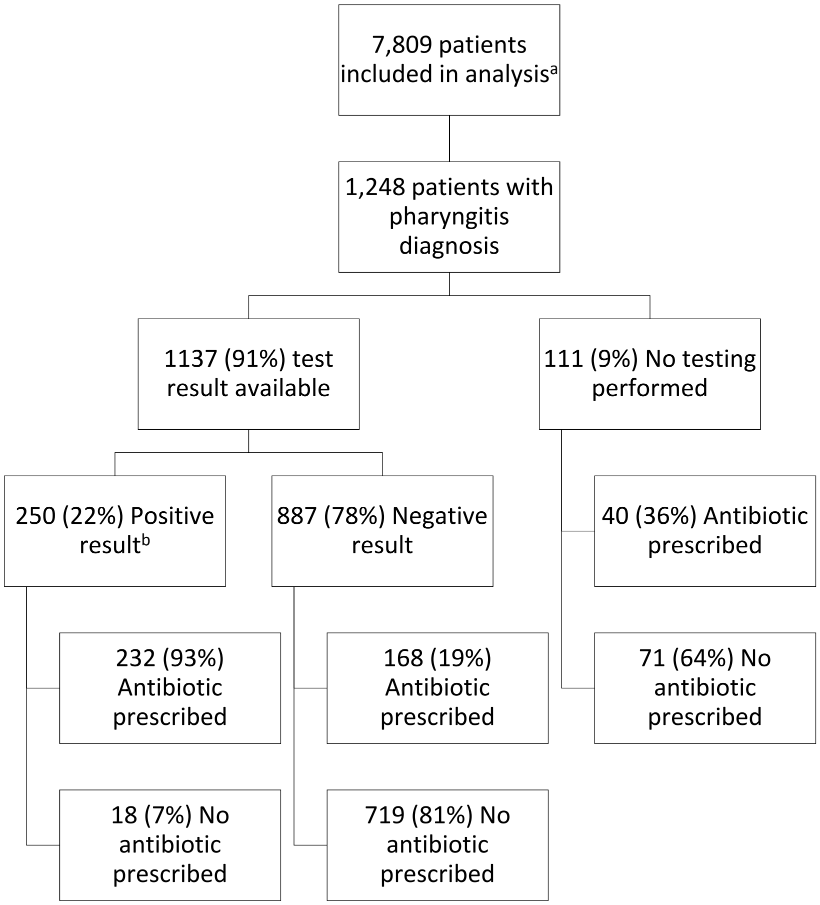

[a] Information on streptococcal testing was available for the 2014-15 season only. One site did not have results from streptococcus testing available and was excluded from this analysis.
[b] Includes positive results from either Group A streptococcus rapid antigen detection test or culture.

© 2018 Havers FP et al. *JAMA Network Open.*
